# Decision coaching using the Ottawa family decision guide with parents and their children: a field testing study

**DOI:** 10.1186/s12911-014-0126-2

**Published:** 2015-02-07

**Authors:** Bryan Feenstra, Margaret L Lawson, Denise Harrison, Laura Boland, Dawn Stacey

**Affiliations:** School of Nursing, Faculty of Health Sciences, University of Ottawa, 451 Smyth Road (Rm 1118), Ottawa, ON K1H 8 M5 Canada; Children’s Hospital of Eastern Ontario Research Institute, Ottawa, ON Canada; Children’s Hospital of Eastern Ontario, Ottawa, ON Canada; Clinical Epidemiology Program, Ottawa Hospital Research Institute, Ottawa, ON Canada

**Keywords:** Children, Parents, Decision-coaching, Patient decision aid, Diabetes

## Abstract

**Background:**

Although children can benefit from being included in health decisions, little is known about effective interventions to support their involvement. The objective of this study was to evaluate the feasibility and acceptability of decision coaching guided by the Ottawa Family Decision Guide with children and parents considering insulin delivery options for type 1 diabetes (insulin pump, multiple daily injections, or standard insulin injections).

**Methods:**

Pre-/post-test field testing design. Eligible participants were children (≤18 years) with type 1 diabetes and their parents attending an ambulatory diabetes clinic in a tertiary children’s hospital. Parent–child dyads received decision coaching using the Ottawa Family Decision Guide that was pre-populated with evidence on insulin delivery options, benefits, and harms. Primary outcomes were feasibility of recruitment and data collection, and parent and child acceptability of the intervention.

**Results:**

Of 16 families invited to participate, 12 agreed and 7 attended the decision coaching session. For the five missed families, two families were unable to attend the session or the decision coach was not available (N=3). Baseline and immediately post-coaching questionnaires were all completed and follow-up questionnaires two weeks post-coaching were missing from one parent–child dyad. Missing questionnaire items were 5 of 340 items for children (1.5%) and 1 of 429 for parents (0.2%). Decision coaching was rated as acceptable with higher scores from parents and their children who were in earlier stages of decision making.

**Conclusion:**

Decision coaching with children and their parents considering insulin options was feasible implement and evaluate in our diabetes clinic and was acceptable to participants. Recruitment was difficult due to scheduling restrictions related to the timing of the study. Coaching should target participants earlier in the decision making process and be scheduled at times that are convenient for families and coaches. Findings were used to inform a full-scale evaluation that is currently underway.

## Background

According to the United Nations Convention on the Rights of the Child, parents are required to hold the ‘best interests’ of children as their primary concern when raising their children [1]. Only when children reach the age of majority can they assume this responsibility for themselves. As children develop skills necessary for decision making, such as abstract thought, problem solving, and inductive and deductive thinking, parents and clinicians should assess the level of involvement children ought to have for various decisions [2,3]. Like adults, children have personal preferences regarding options for different health-related decisions [4,5]. Consequently, unless parents and clinicians actively involve children in a process of shared decision making whereby children, family members and clinicians exchange information on options and treatment preferences to reach an agreement upon a treatment option plan, they risk making choices that lack concordance with the values and preferences of the child [4,6,7].

Children are not consistently consulted or optimally involved in health decisions [3]. Interventions such as decision coaching and patient decision aids improve adult patients’ participation in health decisions, knowledge and decision quality but little is known about their ability to facilitate shared decision making with children [8,9]. Decision coaching is a process of non-directive support by a trained facilitator to help patients develop skills in preparation for decision making with their physician [9]. Decision coaching, often provided by nurses, social workers, or other allied health professionals, alone or in conjunction with patient decision aids, improves knowledge, increases satisfaction, and reduces healthcare costs [9].

Patient decision aids are tools (e.g. pamphlets, videos/DVDs, web-based) that help patients consider treatment options and their outcomes, clarify personal values relating to different management options and outcomes, and proceed through the steps of deliberation and communication with their health care provider [8]. Decision aids reduce decisional conflict in adults, while improving knowledge, the quality of patient participation, and the congruency of patient preferences with chosen treatment options [8]. Given that patient decision aids are not available for all possible health conditions, generic decision aids have also been developed [8,10]. One example of a generic decision aid is the Ottawa Personal Decision Guide, which was developed for use between adult patients and their practitioners. When accompanied by decision coaching, the Ottawa Personal Decision Guide decreases decisional conflict in women considering prenatal testing [10]. The Ottawa Family Decision Guide (OFDG) is an adapted version of the Ottawa Personal Decision Guide that captures both the child and the parents’ perspectives [11].

Despite the evidence for decision coaching and patient decision aids in adult patients, little is known about the use and effectiveness of decision coaching and decision aids in the pediatric context where multiple stakeholders (child, parent(s), and health professionals) are involved. A systematic review revealed that only 5 studies have evaluated interventions to improve children’s participation in health decisions [12]. Interventions included decision coaching with or without a supplemental aid and an educational workshop. Results were mixed. In one study, coaching alone increased child decision making satisfaction and improved parent and child values congruence. The educational workshop increased the overall quality of the decision making process. Two studies used coaching with a supplemental aid and showed no differences in decision making quality or congruence between the child’s preferred and chosen option. However, these interventions primarily involved only the children. Furthermore, none of these interventions are publically available. The objective of this field study was to evaluate the feasibility and acceptability of decision coaching guided by the OFDG with children and parents considering insulin delivery options for type 1 diabetes management.

A decision about type 1 diabetes insulin delivery was selected because it is a common decision that has implications for both the child and parents given the benefits, risks and inconveniences of different insulin delivery options [13]. Options include insulin administered by daily injections or through an insulin pump. Children may be more likely to focus on short term benefits and harms of injections versus pump therapy while parents may be motivated by longer term outcomes [14,15]. In addition to potential inter-personal conflicts that may arise, deciding about the best insulin delivery option may result in decisional conflict or a state of personal uncertainty about the best treatment option for both parent and child.

## Methods

### Study design

A pre-test/post-test field testing study was guided by the Ottawa Decision Support Framework. This framework has three key process elements: a) identification of patient’s modifiable decision making needs; b) intervention designed to address modifiable needs; and c) evaluation of the decision support provided [16]. Field or beta-testing the decision aid with patients and clinicians in real life decision making situations is considered an important step for developing decision aids [17] as it allows the decision aid to be revised based on field testing results. The study received ethics approval from the university and the Children’s Hospital of Eastern Ontario research ethics board.

### Participants and recruitment strategy

Eligible children had type 1 diabetes, were 18 years old or younger, understood and spoke English or French, were able to provide consent, had a guardian who was also willing to participate in the study, and were considering a change in insulin delivery for management of type 1 diabetes. Recruitment occurred in a diabetes clinic in a tertiary children’s teaching hospital. Potential participants were introduced to the study during an insulin pump information session or prior to a scheduled insulin pump assessment. A diabetes nurse educator contacted those who expressed interest in the study by telephone to assess eligibility and explain the study in greater detail. Both children and parents who agreed to participate provided written informed consent.

### Intervention

**Figure 1:**
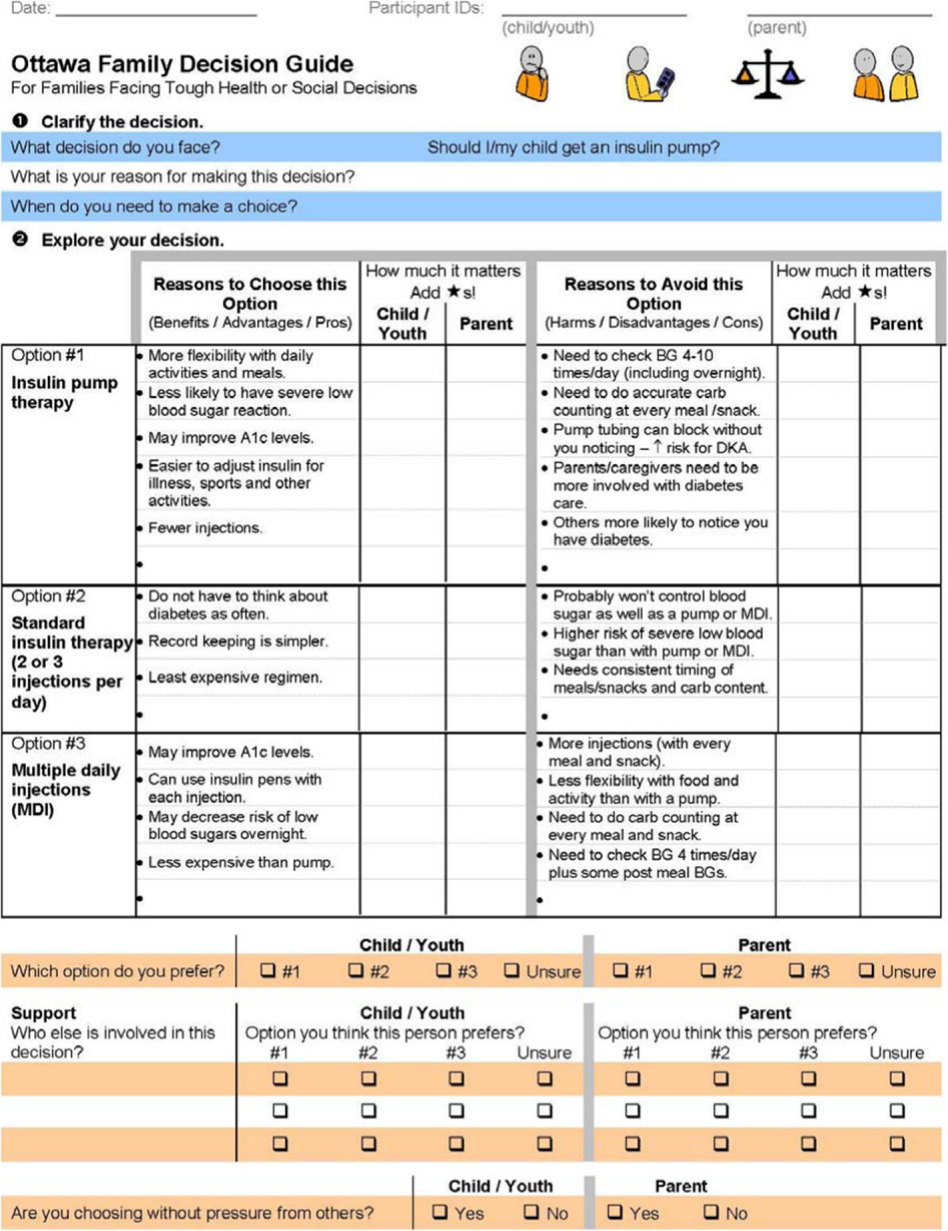
**OFDG pre-populated with insulin options for type 1 diabetes management (page 1).**

**Figure 2:**
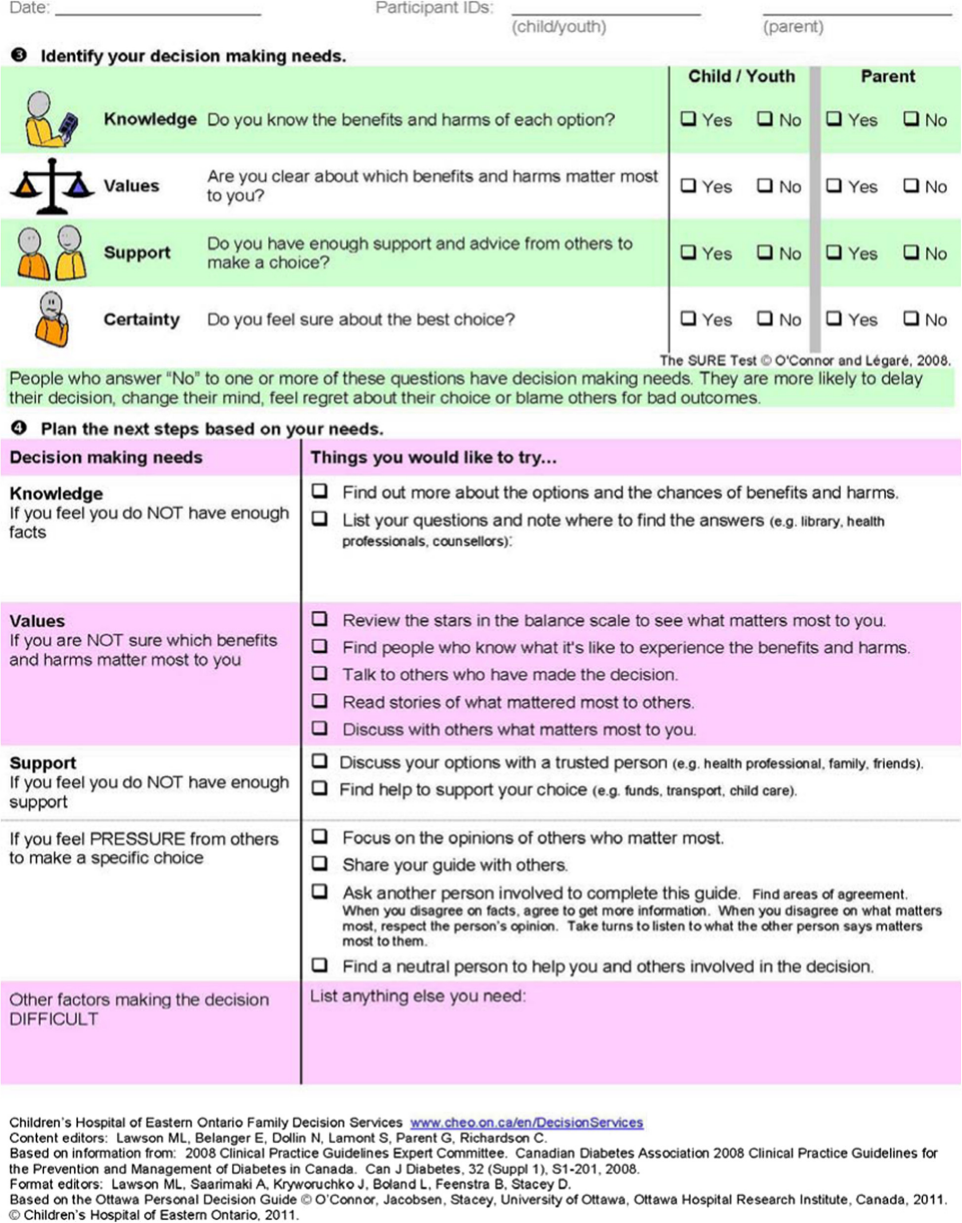
**OFDG pre-populated with insulin options for type 1 diabetes management (page 2).**

The intervention consisted of decision coaching using the OFDG. Decision coaching was conducted by one of two diabetes social workers who had been trained in decision coaching and was part of the local diabetes team. Their role was to help the child and family make the decision explicit, clarify values for benefits and harms of options, identify the child’s preferred treatment option, assess pressures from others, and screen for remaining decision making needs. The OFDG (Figures 1 and 2) provides the decision coach, child, and family a structured guide for working through the decision making process and documenting relevant information. The OFDG was pre-populated with benefits and harms relating to three common options for insulin delivery: insulin pump therapy, standard insulin therapy (2 or 3 injections per day), and multiple daily injections (MDI). Pre-populated items were based on diabetes management clinical practice guidelines and clinical experience [17]. A blank line for each option was provided to allow participants to add other benefits or harms but there was no space for adding other options given these were the only medically reasonable options available. The populated OFDG was peer-reviewed by a Pediatric Endocrinologist and three Diabetes Nurse Educators before being used in this study.

The intervention occurred in a hospital meeting room where confidentiality could be ensured and occurred either immediately following the pump assessment (i.e., the same day), or within 2 weeks of the pump information session. The decision coach explained the purpose of the intervention and proceeded through the steps of the OFDG. The child’s preferences and values were elicited prior to their parent(s) to encourage child involvement and to help avoid biasing the child’s responses. The decision coach facilitated discussions between parent and child regarding agreement/disagreement on values for benefits and harms of the options. A printed copy of the completed OFDG was filed in the hospital chart and provided to the family.

### Procedures

Data were collected at three points during the study: baseline, after decision coaching, and within two weeks of decision coaching. At baseline, children and parent(s) completed the demographic survey, decisional conflict scale [18], and indicated their preferred option [19]. Participants then received decision coaching using the OFDG. Immediately after the intervention the child, parent(s), and decision coach completed the dyadic OPTION scale [20]. Post-decision coaching, families were given copies to take home and instructed to independently complete the decisional conflict scale, acceptability survey, and actual choice. They were given postage paid envelopes to return them by mail within two weeks.

### Outcome measures

Feasibility of the study design was measured by proportion of participants recruited and proportion of missing questionnaire items from children and parents. The other outcomes are consistent with the Ottawa Decision Support Framework and International Patient Decision Aids Standards [16,21,22]. Intervention acceptability was assessed by child and parents’ satisfaction with decision coaching measured by the Genetic Counseling Satisfaction Scale and perceptions of decision coach neutrality [23,24]. This scale has face validity and a reliability coefficient of alpha=0.80 to 0.90 in genetic counselling contexts with adults, however it has not been tested with children [24,25].

Secondary outcomes included child and parent decisional conflict, preferred option, actual choice, values for outcomes of options, and perceptions of involvement in shared decision making. Decisional conflict was measured using the 10-item, low literacy version of the decisional conflict scale having a 3-point response scale of ‘yes’, ‘unsure’, ‘no’ for 10 items [19]. The original scale has good reliability (0.86) and internal consistency (0.78-0.92) in adults, was validated with parents in the pediatric context, and has been used with youths making health-related decisions [26,27].

Preferred option was measured using the 15-point Choice Predisposition Scale that demonstrates a patient’s inclination toward a given option, with a score of 1 indicating a strong preference for option A, 15 indicating a strong preference for option B and the centre of the scale indicating no preference [19]. With three options for insulin delivery, this question was repeated three times, comparing options A with B, B with C, and C with A. This scale has a test-retest coefficient >0.90 [27].

Parent, child and decision coach perceptions of decision making involvement were measured using the 12-item dyadic OPTION scale [20]. This scale is valid and has moderate inter-rater reliability (ICC 0.77) when used by physicians and their adult patients [28]. To fit within the role of decision coaches, three items were modified (i.e., sources of information discussed, ways to manage health problem discussed, preferred option chosen) and the revised version was approved for use by the original author (GE). Values for outcomes of options were measured using a balance scale method included in the OFDG. This 5-point scale helps participants evaluate the desirability of benefits and harms relating to options. Test-retest coefficient was 0.79 to 0.91 [19].

### Analysis

Data were entered into an Excel database and analyzed descriptively. Findings were described for children and parents recruited at earlier versus later stages of decision making.

## Results

### Participants

**Figure 3:**
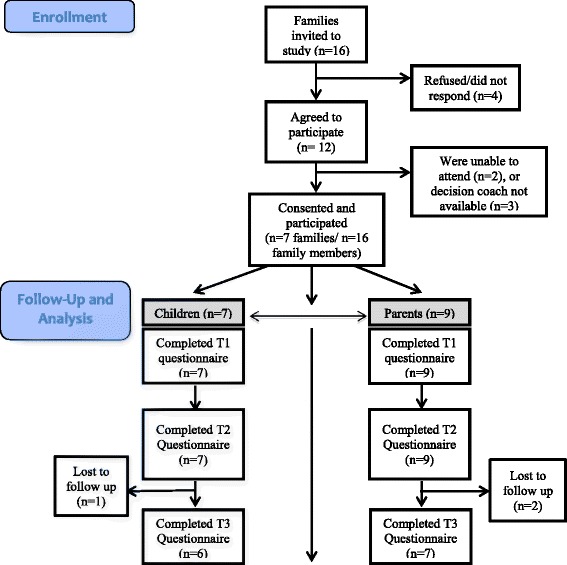
**Diagram of study flow.** Families were invited to participate in the study prior to a scheduled insulin pump assessment or at an insulin pump information evening and attended either shortly after the pump assessment or within 2 weeks following the information evening. Seven families, including 16 family members (7 children and 9 parents), consented and participated in the study.

Of 16 families approached to participate, 12 agreed (75% response rate). Of the 12 families, 7 consented and were able to attend the decision coaching session (58% retention rate; Figure 3). Reasons for not receiving decision coaching were that families were unable to attend (n=2) and the decision coach was unavailable (n=3).

The 7 families were comprised of 16 family members (7 children and 9 parents) who consented and participated in the study. One parent participant offered to sign consent after the intervention, therefore, she was not included in the analysis. Two families had two participating parents and 5 had one parent participate in the decision coaching intervention. Four children were male and 3 were female with an age range of 9 to 17 (Table 1). Four parents were male and 5 were female with an age range of 29 to 54 and an education ranging from some high school to university graduate. Of 7 families, 2 were considering their options and 5 preferred insulin pump therapy at baseline.

**Table 1:** **Characteristics of child–parent dyads (n=7)**

**Demographic characteristics**	**Children**	**Parents**
***Children***	**(n=7)**	**(n=9)**
Age (in years)	Median: 15	Median: 44
	(Range: 9,17)	(Range: 29,54)
≤9	1	
10-14	2	
15-19	4	
Sex:		
Male	4	4
Female	3	5
Primary Language:		
English	6	8
French	1	1
Duration of Type 1 Diabetes:		
>1 month to 6 months	1	
>6 months to 1 year	2	
>1 year to 5 years	2	
>5 years	2	
Education:		
*Child- Grade Level*		
≤4	1	
5-8	2	
9-12	4	
1^st^ Year University/College	0	
*Adult- Highest Education*		
Some High school		3
High school diploma		1
Trade cert./diploma		0
University-undergraduate degree		3
University-graduate degree		2
Marital Status:		
Married/Common-law		8
Divorced/Separated		1

### Feasibility

All 9 parents and 7 children completed the baseline and post-coaching questionnaires. Of 16 participants, 13 (representing 6 families) returned the questionnaire two weeks post-coaching (81% response rate). Missing questionnaire items were 5 of 340 for children (1.5%) and 1 of 429 for parents (0.2%). Parents and children were able to complete questions on all the instruments used for secondary measures in the study. For 2 of the 7 families the coach suggested a specific option. Four of seven decision coaching sessions were timed. The median time was 35 minutes (range 21–38 minutes).

### Acceptability

Of the 7 children, most rated the coach as being concerned about their well-being, would recommend it to others, and thought it prepared them for follow-up with their health care provider (5 or 6 out of 7 children) (Table 2). Some children indicated that the coach understood the stresses they felt, helped identify what they needed to know to make a decision, and felt the session was the right amount of time (3 or 4 out of 7 children). An audit of the completed OFDG showed that the child’s ratings on the importance of values, and the influence of others involved in the decision were not always concordant with their parent(s). However, most children did not think the OFDG was valuable or helpful. Of the 9 parents, most agreed that the coach understood the stresses they felt, helped to identify what they needed to know to make a decision, felt better about the decision after coaching, thought the coach was truly concerned about their well-being, helped them come to a preferred option, felt prepared for their follow-up visit to the health care provider and would recommend it to others (7 or 8 out of 9 parents). Only some parents thought the decision coaching was valuable and the right length of time (4 or 5 out of 9 parents).

**Table 2:** **Acceptability of decision coaching session**

**Items with response options**	**Child**	**Parent**
	**(n=7)**	**(n=9)**
The decision coach seemed to understand the stresses I was facing.		
Agree strongly/somewhat	3	8
Neutral	2	0
Disagree somewhat/strongly	0	0
No response	2	1
The decision coach helped me to identify what we needed to know to make decisions about what would happen to me.		
Agree strongly/somewhat	4	8
Neutral	0	0
Disagree somewhat/strongly	2	0
No response	1	1
I felt better about my decision after meeting with the decision coach.		
Agree strongly/somewhat	2	8
Neutral	1	0
Disagree somewhat/strongly	2	0
No response	2	1
The decision coach was truly concerned about my well-being.		
Agree strongly/somewhat	5	7
Neutral	0	1
Disagree somewhat/strongly	1	0
No response	1	1
The decision coaching session was valuable to me.		
Agree strongly/somewhat	1	5
Neutral	0	0
Disagree somewhat/strongly	3	1
No response	1	2
How helpful was the decision coaching in helping you come to a preferred option?		
Very/somewhat helpful	2	7
A little helpful	3	1
Not helpful	1	0
Not answered	1	1
Would you recommend decision coaching to others facing the same decision?		
I would definitely/probably recommend it	5	8
I would probably/definitely not recommend it	1	0
No response	1	1
The decision coaching session was about the right length of time.		
Agree strongly/somewhat	3	4
Neutral	0	4
Disagree somewhat/strongly	3	0
No response	1	1
Did this session prepare you for a follow up with your health care provider?		
Yes	6	8
Unsure	1	0
No	0	1

Children and parents recruited earlier in the process (e.g. at the insulin pump information session) rated the coaching as more acceptable and the right length of time. As one participant noted *“…I think ‘decision coaching' could be helpful to those who are truly undecided, but for us it wasn't too helpful as we had made up our minds about going on a pump”* (parent, age 44). Two other children echoed this sentiment stating *“…I allready (sic) made my desison (sic) about wich (sic) I want. I want a pump now”* (child, age 9) and*, “My choice was all ready (sic) made”* (child, age 15). Parents of a family recruited later (e.g., at the insulin pump assessment) said: *“the session was a little too long for our nine year old daughter”.*

## Discussion

This field study informed the acceptability of the intervention and feasibility of a study designed to evaluate decision coaching using the OFDG with parents and children. While decision coaching with children and parents was feasible to provide and measure, the study encountered challenges with the overall participation rate due to limited decision coach availability and extra time required for children and parents to attend the session. The intervention was rated as more acceptable by parents and children who were looking for information on insulin pump therapy compared to those who had already attended a pump assessment.

### Feasibility

Evaluation of decision coaching using the OFDG was deemed feasible due to the high rate of completed questionnaires and low rate of missing data. Our study recruitment, however, was slower than rates noted in adult patient decision coaching studies [9]. According to a recent systematic review, decision coaching had not been previously implemented in pediatric clinical settings making recruitment comparisons difficult [12]. Our recruitment rates appear to be related to offering coaching on the same day that families had another scheduled appointment. Many families were not willing to extend their appointment to participate in decision coaching. Furthermore, decision coaches were often unavailable during this time. These recruitment challenges were resolved in the full-scale study by providing each coach with a weekly one hour block and scheduling families to come specifically for coaching.

Decision coaches are trained to be non-directional [9]. According to two families, one of the two decision coaches involved in our study recommended a specific option. This may have been due to a potential familiarity of the decision coaches with diabetes management and/or these families; thereby, making it more difficult for them to remain non-directive when discussing the treatment options and their suitability for different families [29]. This issue was addressed directly with the decision coaches during the study by reinforcing the importance of remaining non-directive during the coaching process.

### Acceptability of decision coaching with the OFDG

Although parents generally responded positively to the decision coaching using the OFDG, the acceptability was more variable among the children. Parents and children who were earlier in their decision making rated the decision coaching as being more acceptable compared to those recruited at the pump assessment. Attendance at an insulin pump assessment is required for initiation of insulin pump therapy through this diabetes clinic and at the time of this study, families were waiting 12–18 months for pump assessments, during which, many may have solidified their intention to start pump therapy. It is likely, therefore, that by the time children and parents attended the pump assessment they had already formed a preference for the insulin pump option. In contrast, families who participated after attending the pump information evening were likely at an earlier stage in decision making.

Variations in satisfaction ratings among children may also be in part, age-related. Although some studies suggest children want to be involved in decision making, children’s willingness and ability to participate in decision making may be tied to factors such as level of clarity regarding legal rights to participation, parental influences, and experience with their health condition [3,30,31]. Such factors may not be present at younger ages, thus influencing a child’s willingness to participate. For example, younger children (age 8, 9) think they should participate in health decisions at 14 or 15 years of age, while older children (age 13, 14) believe participation can occur as young as age 13 [30]. However, children with chronic illnesses such as diabetes are more knowledgeable about their condition and they may be able to participate in condition-related decisions earlier than their healthy peers [32].

Field testing of decision coaching using the pre-populated OFDG with children and their parents actively considering the decision led to three changes to the intervention [33]. As mentioned earlier, decision coaches were reminded to be non-directive during their coaching sessions. Second, the OFDG was changed to present the option of standard insulin therapy (2 or 3 injections per day) first, followed by multiple daily injections and then insulin pump therapy, given that children are typically on standard insulin therapy when considering other options. Third, decision coaching is now offered to families immediately after they express interest in changing their insulin delivery method, at times mutually convenient for the families (including both parents) and decision coaches. Pump assessments are then scheduled after decision coaching is completed for those who have decided that they want to proceed with initiating pump therapy.

### Patient and family centered approach

Decision coaching can be provided internally (by members of the local health team) or externally (by independent decision coaches) [29]. We purposely structured this intervention so that it was provided by internal staff members of the local diabetes team, with the goal of enhancing sustainability and aligning it with the Patient and Family Centered Care model promoted at the children’s hospital. Internal delivery of decision support may allow greater family-centeredness because patients have timely access to additional decision support within the care pathways of their diabetes team. Families may also experience increased trust with known health professionals who are familiar with them and have content expertise.

The success of pediatric decision support where multiple stakeholders are involved (child, and one or more parents/guardians) may be more effective if all relevant individuals are present during decision coaching. In this study, both parents were present at the decision coaching with four of the seven families (though not all parents participated in completing questionnaires). For the remaining three families, only the mother was present. Although it may be ideal to have all relevant stakeholders present at the decision coaching session, scheduling a time when both parents can attend may be unrealistic.

### Limitations

There are four key limitations that should be considered when interpreting the results of this study. First, study recruitment was hindered by difficulties coordinating availability between the decision coach and family. To address this issue, decision coaching needs to be incorporated into the social worker’s responsibilities within the diabetes team and protected time for decision coaching needs to be ensured. Second, none of the instruments objectively measured child or parent involvement. Although children completed the dyadic option scale as a surrogate indicator of their level of involvement, ideally the coaching sessions should be video or audio recorded to facilitate a more objective measure. Third, self-selection bias may have occurred as only those families motivated and willing to participate in decision coaching agreed to participate in the study. However, we were able to include participants of both sexes, and of variable age, diabetes duration, and education levels. Finally, this approach to evaluating decision coaching using the OFDG will not allow us to separate the effects of coaching from the effects of the OFDG.

### Practice implications

This study is unique in that it presents an approach for incorporating the child’s perspective into the decision making process. Results suggest that treatment choices can successfully be made by parents together with their child. Health professionals should consider how decision making might best be facilitated among multiple stakeholders (children, parents, and clinicians) and consider employing interventions that successfully meet the needs of all those involved [3,34].

The OFDG populated with evidence from clinical practice guidelines for insulin delivery options demonstrated promise as a decision support intervention, especially as an adjunct to decision coaching [10]. Given that little is known about the effects of decision aids when used by children, further evaluation of the OFDG decision aid is required to determine the impact on decisional conflict and other decision making outcomes [8]. The acceptability ratings are encouraging for future testing of the OFDG with this population. Since the OFDG is a generic decision aid, it can be used for other pediatric decisions. This is particularly important given the limited availability of condition specific decision aids for children [11].

## Conclusion

This field study showed that decision coaching with the OFDG was acceptable to parents and children considering insulin options for type 1 diabetes management and was feasible to measure. However, there were challenges with recruitment mostly due to time constraints of decision coaches and families. The intervention requires further evaluation to determine its impact on parent and child outcomes but this requires targeting families at an earlier stage in the decision making process. Future research could also explore the use of decision coaching with the OFDG in other clinical areas where decision support may address decision making needs of children and parents.

## Abbreviation

OFDG: Ottawa family decision guide
